# Progressive influence of body mass index-associated genetic markers in rural Gambians

**DOI:** 10.1136/jmedgenet-2014-102784

**Published:** 2015-04-28

**Authors:** Anthony J Fulford, Ken K Ong, Cathy E Elks, Andrew M Prentice, Branwen J Hennig

**Affiliations:** 1 MRC International Nutrition Group at LSHTM, UK & MRC Unit, The Gambia; Department of Epidemiology and Population Health, London School of Hygiene & Tropical Medicine, Keppel Street, London, UK; 2 MRC Epidemiology Unit, University of Cambridge, School of Clinical Medicine, Box 285 Institute of Metabolic Science, Cambridge Biomedical Campus, Cambridge, UK

**Keywords:** genetic risk score, BMI, Gambia, longitudinal analysis, cross-sectional analysis

## Abstract

**Background:**

In populations of European ancestry, the genetic contribution to body mass index (BMI) increases with age during childhood but then declines during adulthood, possibly due to the cumulative effects of environmental factors. How the effects of genetic factors on BMI change with age in other populations is unknown.

**Subjects and methods:**

In a rural Gambian population (N=2535), we used a combined allele risk score, comprising genotypes at 28 ‘Caucasian adult BMI-associated’ single nucleotide polymorphisms (SNPs), as a marker of the genetic influence on body composition, and related this to internally-standardised z-scores for birthweight (zBW), weight-for-height (zWT-HT), weight-for-age (zWT), height-for-age (zHT), and zBMI cross-sectionally and longitudinally.

**Results:**

Cross-sectionally, the genetic score was positively associated with adult zWT (0.018±0.009 per allele, p=0.034, N=1426) and zWT-HT (0.025±0.009, p=0.006), but not with size at birth or childhood zWT-HT (0.008±0.005, p=0.11, N=2211). The effect of the genetic score on zWT-HT strengthened linearly with age from birth through to late adulthood (age interaction term: 0.0083 z-scores/allele/year; 95% CI 0.0048 to 0.0118, p=0.0000032).

**Conclusions:**

Genetic variants for obesity in populations of European ancestry have direct relevance to bodyweight in nutritionally deprived African settings. In such settings, genetic obesity susceptibility appears to regulate change in weight status throughout the life course, which provides insight into its potential physiological role.

## Introduction

Genome-wide association studies (GWAS) in populations of European ancestry have identified more than 50 loci that are robustly associated with adult body mass index (BMI). The study by Zhu *et al*^1^ gives a comprehensive summary of recent GWAS on obesity-related traits conducted in different populations. Far fewer data are available regarding populations of different ethnic backgrounds, especially those living in nutritionally-deprived settings. Most studies in Asian populations have replicated BMI ‘risk’ variants, though lower effect sizes have been reported.^1^ ^2^ Studies in populations of African ancestry have provided inconsistent results regarding the contribution of genetic variation to BMI and obesity.^3–5^ In a recent meta-analysis, Monda *et al*^6^ demonstrated that out of 36 predominantly ‘Caucasian BMI loci’, five individually reached genome-wide significance in African Americans and two novel loci were identified through their analysis. Such discrepancies in findings from different studies are thought to be due, in part, to variation in the genetic architecture of different populations. Given that populations of African origin are characterised by greater genetic heterogeneity (manifested in lower linkage disequilibrium (LD) and smaller haplotype blocks), difficulties can arise in replicating signals identified in populations of European ancestry. On the other hand, this may lend an advantage for fine-mapping putative causal variation and the identification of novel loci.^7^ ^8^ For example, a recent study by Gong and colleagues was able to narrow the signal to a substantially smaller number of associated single nucleotide polymorphisms (SNPs) in several BMI-related loci in African Americans.^9^

Much data on the genetic basis of BMI and obesity published to date has concentrated on adult BMI; however, these variants have also been shown to influence weight gain and obesity risk in childhood, but not birthweight (BW).^1^ ^10–13^ Such studies have reported that the effect size of adult BMI variants on bodyweight increases during childhood and plateaus during adolescence and adulthood. Lack of associations with adult weight gain could reflect developmental stage-specific effects of genetic obesity mechanisms or the relatively greater influence of accumulated environmental factors.

Given the small effect sizes seen for individual markers, many studies have analysed combined allele scores in order to evaluate more effectively the cumulative influence of adult obesity susceptibility on childhood growth and other traits.^10^ ^11^ ^13–17^ Employing a composite score of risk alleles maximises statistical power, reduces the pitfall of multiple testing, and widens the generalisable nature of the findings.^18^

Healthy growth is particularly important in children in the world's poorest nations where malnutrition and infection pose a double burden. In sub-Saharan Africa growth faltering remains a major public health issue.^19^ ^20^ Understanding the nutritional and genetic basis of growth failure to identify critical windows of opportunity for interventions is thus essential. We took advantage of our well-characterised Gambian core-village population, with detailed anthropometric measures in particular during early infancy, to assess the cumulative effect of ‘Caucasian adult BMI-associated’ SNPs on anthropometric outcomes in this rural African setting. We adapted a set of 32 SNPs described by Speliotes and colleagues,^21^ replacing markers that are located in different haplotype blocks in Europeans and Africans with surrogate SNPs that are in high LD with the original SNPs. This was done in order to increase the likelihood of tagging the associated variant in our Gambian population. We interrogated our data both cross-sectionally and longitudinally, focusing on early growth (0–2 year) and growth to adulthood (2–20 year), and further assessed the predicted increase in z-score for weight-for-height (zWT-HT) per risk allele score as a function of age.

## Methods

### Study sample

Individuals resident within the three ‘core villages’ around the Medical Research Council (MRC) field station in Keneba (West-Kiang, The Gambia) were considered eligible for the present study. An antenatal/maternity programme has been running in these villages (Keneba, Kantong Kunda, and Manduar) since 1974. Records and census data going back to the 1950s is available through the Keneba database maintained by the MRC International Nutrition Group. This includes demographic data on ∼13 300 individuals and was used to establishment pedigree relationships. This population comprises socially homogenous, rural subsistence farmers, the majority of whom are Mandinka (self-reported ethnicity). The adult age lower cut-off was defined as 20 years.^22^ The attrition of available data at each stage of the study is summarised in table 1.

**Table 1:** Cumulative effect of 28 risk alleles on anthropometric measures

Age	Outcome	N	Coefficient*	95% CI	p Value
Cross-sectional analysis					
Birth	zBW	1237	0.008	−0.011 to 0.026	0.422
2 years†	zWT	1820	0.012	−0.003 to 0.027	0.134
2 years†	zHT	1820	0.009	−0.006 to 0.025	0.246
≤20 years	zWT-HT	2211	0.008	−0.002 to 0.017	0.108
Adults‡	**zWT**	1426	**0**.**018**	0.001 to 0.035	**0**.**034**
Adults‡	zHT	1426	0.004	−0.012 to 0.021	0.609
>20 years	**zWT-HT**	1426	**0**.**025**	0.007 to 0.044	**0**.**006**
Adults‡	zBMI	1426	0.016	−0.001 to0.034	0.060
Longitudinal analysis					
0–2 years	WT growth	1248	0.001	−0.009 to 0.012	0.810
0–2 years	LG growth	1214	0.005	−0.000 to 0.011	0.064
2–20 years	zWT change	872	0.010	−0.016 to 0.035	0.455
2–20 years	zHT change	872	0.006	−0.020 to 0.032	0.666

*Coefficients indicate change in z-scores per allele (cross-sectional) or change in z-scores per allele per year (longitudinal).

†Nearest observation to 2 years of age (>1.5 and <2.5 years).

‡First measurement at adult age (>20 years).

BMI, body mass index; BW, birthweight; HT, height; LG, length; WT, weight; zBMI, BMI z-score; zBW, BW z-score; zHT, height-for-age z-score; zWT, weight-for-age z-score; zWT-HT, weight-for-height z-score.

Results significant at the p<0.05 level are indicated in bold.

This study was approved by the joint Gambia Government/MRC Ethics Committee (SCC/ECL2009.61) and all subjects and/or legal guardians provided written, informed consent.

### Growth measures

Anthropometric measures comprised z-scores for birthweight (zBW), zWT-HT, weight-for-age (zWT), height-for-age (zHT), as well as body mass index (zBMI). A total of >43 000 weight and length/height measurements were available for analysis. All measures were taken by trained midwives or field staff with regularly calibrated equipment. BW was recorded to the nearest 10 g within 72 h of birth. Weight was recorded to the nearest 10 g and height to the nearest 0.1 cm. Z-score calculations are described under the statistical analyses section below.

### Genetic analyses

DNA was extracted from venous blood samples collected in 2002–2003 using a standard salting-out method according to the protocol of the DNA Bank at the MRC Laboratories in The Gambia.^23^

We designed a single 30-plex assay analogous to the set of 32 ‘Caucasian adult BMI-associated’ SNPs reported by Speliotes *et al*.^21^ If the original SNP was shown to locate in a different haplotype block based on HapMap Yoruba (YRI) compared to European (CEU) data, an alternative marker was selected using a cut-off of r^2^ >0.8 with the original SNP. Similarly, because some SNPs had a minor allele frequency (MAF) <1% in YRI, an alternative and/or additional marker was selected as described and genotyped. For three of the original SNPs no suitable surrogate SNP was identified. The total number of SNPs screened was 30, two of which were monomorphic in Gambians; see online supplementary table S2 for details. Genotyping was performed at the MRC Epidemiology Unit, Cambridge on the Sequenom iPLEX platform (Sequenom, San Diego, California, USA) as previously described.^10^ Hardy-Weinberg equilibrium (HWE) was tested based on one randomly selected representative per sibship (defined as having the same mother).

The BMI-increasing allele for each SNP is indicated in online supplementary table S2. One variant (rs4836133 in ZNF608) is tri-allelic and was recoded as bi-allelic according to the number of BMI-increasing alleles. For individuals with missing genotypes at only five or fewer SNPs, data were imputed using the mean number of BMI-increasing alleles for that SNP (ie, twice the allele frequency). A combined genetic score was then calculated for each individual, comprising the cumulative number of BMI-increasing alleles across all valid genotypes.

### Statistical analyses

Because the study covered a wide range of ages, we calculated internally calibrated z-scores for all anthropometric measures. We estimated several growth parameters for each individual: zBW at birth; zWT and zHT at 2 years (nearest measurement >1.5 and <2.5 years); and zWT, zHT and zBMI in adults (using first measurement at adult age (>20 years)). For zWT-HT in children (≤20 year olds), and in those >20 years of age, we used all available data points, taking into account between-individual and between-family variation in mixed effect models (see below). BMI was calculated for adults only as weight/height^2^ (kg/m^2^; see online supplementary table S1). Since BMI is strongly age-dependent below the age of 18 years, we deemed internally calibrated zWT-HT as the best suited measure of body composition in our population covering a wide age range from birth to older adults.

Internal z-scores and growth measures were calculated as previously described.^24^ Briefly, zWT, zHT and zBMI were calculated for particular age-sex groups using the formula z-score=(x-mean)/SD, where x is an individual's measurement and mean and SD are the sample mean and standard deviation of the measurements within the age-sex group concerned. In the case of WT and HT the measurements were logged first in order to reduce skewness. The same formula was used for zWT-HT except that this variable had to be calculated for measurements at all ages so the mean and SD were calculated as (sex-specific) functions of age. These were derived from regression models fitting log(WT) to polynomials of age and HT and the squared residuals to polynomials of age.

Longitudinal growth analyses were separated into two age periods, between 0–2 years, and between 2–20 years. Infant growth was derived for those individuals providing at least five measurements, with at least one in each of the first and second years of life and where growth per year could be estimated with reasonable accuracy (defined as SE of change in z-score per year <0.4). The internal z-score for each measurement was regressed on age separately for each child. The birth to 2 year growth parameters are the coefficient for age from these regression models. For weight and height growth between the ages of 2–20 years we again calculated internal z-scores (both zWT and zHT) for the anthropometry measurement taken closest to 2 years of age, provided it lay between 1.5 and 2.5 years, and for the first adult measurement (above 20 years). The 2–20 years growth parameter is given by the difference between these two z-scores.

We related the combined genotype score as a linear variable to zWT, zHT and zBMI using random effects regression (generalised least squares) with the higher level of variance given by clusters defined by a mother and her offspring (whether by the same or different fathers). In each analysis we controlled for village of residence and year of birth (to adjust for possible secular changes) by fitting these respectively as categorical and continuous linear terms in the model. For zWT-HT, a similar analysis was performed using a multilevel mixed effects model fitting three levels of variance: between family, between individuals within family, and between observations on the same individual. To estimate how the effect of the genetic score on zWT-HT changed as a function of age, we added to this model terms for the interactions between the genetic score and cubic polynomials for age (fitted as three orthogonal polynomials).

## Results

### Study population

Demographics and summary statistics of the study population are shown in online supplementary table S1.

The sex distribution varied slightly by age group, but ranged between 53–69% females (data not shown). In the current sample, 114/2535 were of unknown ethnicity or non-Mandinka (self-reported ethnicity, see online supplementary table S1). As >95% of individuals were Mandinka, we did not stratify the study cohort by ethnic groups. One of each of nine twin pairs was randomly excluded.

The data analysed were derived from ∼43 250 measurements for weight and length/height in 2535 individuals genotyped in this study, with each individual contributing on average 17 observations (range 1–58), with routinely up to 12 measurements in the first 2 years of life and less frequent measurement thereafter. For 19 individuals either weight or height information was missing. Mean (±SD) BW was 2.94±0.41 kg and mean adult BMI was 21.18±3.01 kg/m^2^. Mean (±SD) adult WT and HT were 58.7±9.0 kg and 1.70±0.069 m for males, and 54.6±9.2 kg and 1.59±0.061 m for females.

### Genetic data

Call rates for all 30 SNPs were 95% or above. Two SNPs (rs10938397 in GNPDA2, and rs13107325 in SLC39A8) were monomorphic in our Gambian sample and were therefore excluded from the analyses. The remaining 28 SNPs passed HWE criteria (p<0.0019, representing p=0.05 corrected for 28 tests).

Data on 74 individuals were excluded from analyses due to missing genotype data on six or more (>20%) of the 28 SNPs. In the remaining sample of 2516 individuals, missing genotypes were imputed using the mean number of BMI-increasing alleles for each variant; the missingness ranged between 0.0–2.9% across the 28 markers included in the analysis (see online supplementary table S2).

### Associations with body size and growth

**Figure 1:**
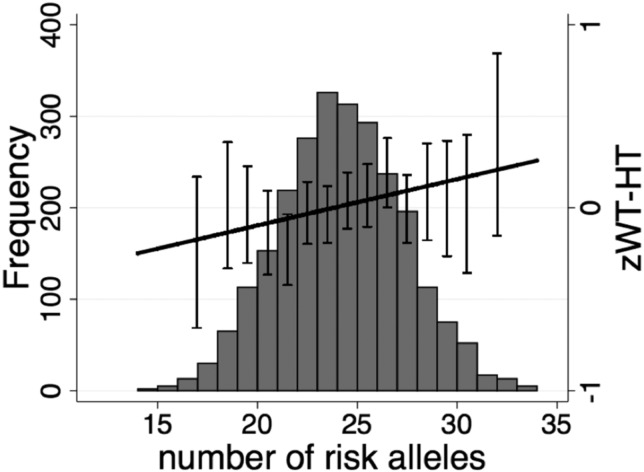
Cumulative risk allele effect on adult z-score for weight-for-height (zWT-HT). Distribution of the genetic predisposition score and cumulative effects of the risk alleles from the 28 variants on adult zWT-HT (N=1426). Mean (±SE) values for zWT-HT is also shown. For the purpose of these graphs no correction for village or year of birth was applied.

In the cross-sectional analysis, the combined allele score across all 28 risk alleles was positively associated with adult zWT (0.018 SDs per allele, CI 95% 0.001 to 0.035; p=0.034) and zWT-HT (0.025, CI 95% 0.007 to 0.044; p=0.006), while a trend towards association were seen for zBMI (p=0.060) (table 1 and figure 1), but not for adult zHT. No genetic score association was seen in the cross-sectional analysis for size at birth, at 2 years, or in individuals ≤20 years of age or for longitudinal changes in zWT or zHT between 0–2 years or between 2–20 years (table 1).

**Figure 2:**
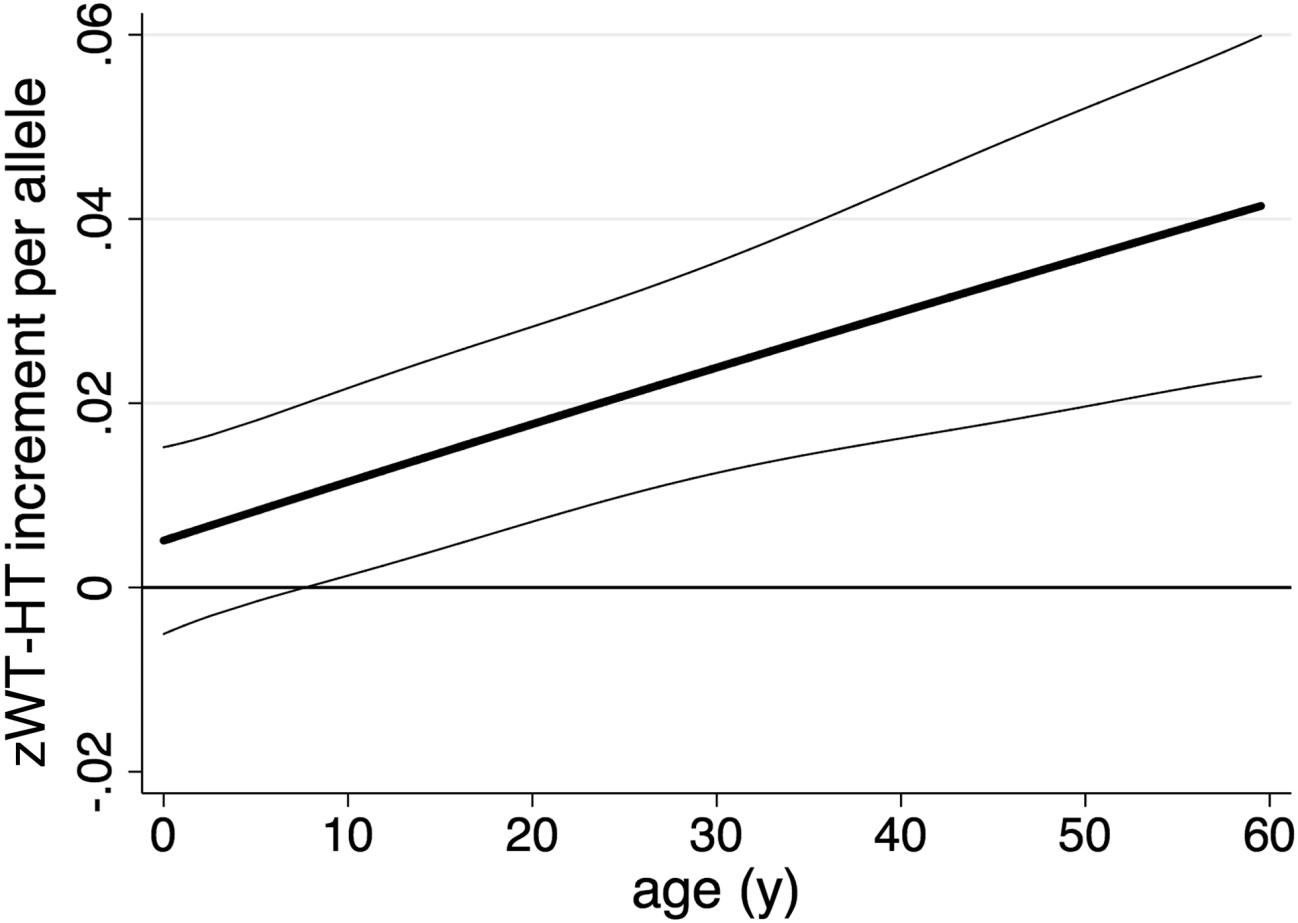
Variation in genotypic effect on z-score for weight-for-height (zWT-HT) with age. The predicted increase in zWT-HT per allele as a function of age is plotted, indicating that the effect of the genetic predisposition score increases throughout life. The data comprise 43 235 measurements in 2513 individuals. Thick line=predicted genotypic effect on zWT-HT with age predicted from models with a cubic function of age; thin lines=95%CI.

We further assessed the interaction between age and the genetic score on zWT-HT. We estimated the increase in zWT-HT per allele as a cubic function of age (figure 2); this showed that the effect of the genetic score on zWT-HT increased linearly with age (linear age-interaction term: 0.0083 z-scores/allele/year; CI 95% 0.0048 to 0.0118, p=0.0000032; quadratic and cubic age-interaction terms were not significant).

## Discussion

This study showed that common genetic variation that is robustly associated with obesity risk in populations of European (and Asian) origin also affects the adult weight status (WT-HT) of lean Africans (rural Gambians) living in a nutritionally deprived environment. A cumulative risk score of 28 ‘BMI’ risk alleles was employed to maximise statistical power, to reduce multiple testing, and to represent a generic index of genetic obesity susceptibility.^10^ ^11^ ^13–18^ We acknowledge that a composite risk score does not allow any inference regarding the possible differential contribution of individual SNPs. However, we previously investigated 16 FTO SNPs in our study population and did not observe individual associations with body mass.^25^

Compared to similar data employing cumulative risk scores, the effect sizes in our study (ie, 0.018 to 0.025 of a z-score per risk allele on adult weight and WT-HT) amounted to approximately one third to one half of that seen in populations of European ancestry and living in affluent environmental conditions using a score of 32 SNPs (0.17 kg/m^2^ per allele).^21^ Of the recent studies including African American samples, some support reduced effect sizes,^18^ while others report comparable results to those seen in populations of European origin.^22^ However, the directional consistency across all these different populations indicates evidence for shared genetic influences on BMI and other anthropometric traits across the whole spectrum of ethnicity and nutritional availability.

Recent publications in non-African origin populations suggest that the genetic contribution to BMI strengthens during childhood and then gradually weakens with age in adults.^15^ ^17^ ^24^ However, others have not seen such changes in adiposity-related traits from adolescence to adulthood.^26^ Furthermore, a recent study suggests that the influence of susceptibility variants has increased during the obesity epidemic, at least in settings where changes in secular trends occur, for example, due to increases in nutritional availability.^13^ Despite significant measures to improve health outcomes, for example, via vaccination programmes, the prevalence of childhood growth deficits remains a major public health problem in sub-Saharan Africa (eg, stunting is generally stagnant at about 40% though it has reduced to 20% in our population).^19^ ^27^ Studies across different low- and middle-income countries have shown that in these regions children are born small and growth faltering, especially in length/height, starts soon after birth and continues for the first 2 years of life. Such analyses have been important in developing the concept that the time between conception and 2 years of age represents an optimal ‘window of opportunity’ within which growth-promoting nutritional interventions should be focused—the first 1000 days.^28^ We thus stratified our data into two groups, representing changes in the first 2 years of age (0–2 years) and from 2 to 20 years. This analysis did not reveal an effect of cumulative risk alleles on changes in zWT or zHT change between the ages of 2 and 20 years, or in length or weight growth between the ages of 0 and 2 years (although there was a trend to a positive association with length growth in children <2 years old). Further analysis revealed a pronounced interaction with age, with significant association seen between genetic risk score and zWT-HT in adults (>20 years), but not in children. Furthermore, while fitting a cubic function to capture the age effect and its interaction with genotype allows for a reasonable degree of flexibility in the shape of the relationship, there was surprisingly clear evidence that the effects of genetic susceptibility accumulate linearly with age (figure 2).

Several reasons are likely to contribute to these differences between our findings and similar genetic studies across different populations. We studied lean Gambians; among the adults within our population (N=1426), only 4.6% are overweight and 0.8% are obese when applying standard BMI cut-off of 25–30 and >30, respectively. Our population represents a lower and narrower range in weight status compared to populations of European ancestry living in affluent conditions, which could contribute to lower effects sizes compared to populations with wider ranges of measures and with phenotypes at the (extreme) upper end of the obesity scale. Furthermore, the nutritionally deprived rural Gambian environment may have directly reduced the influence of BMI-increasing genetic variants; even in other nutritionally richer settings, stronger genetic effect sizes are observed within subgroups who lead more obesogenic lifestyles^29^ and in settings with increasing nutritional availability, leading to secular trends in the obesity epidemic.^13^ Furthermore, our analysis focused on polymorphisms robustly associated with BMI in populations of European origin. Such SNPs may not be ideal to assess the transferability of association signals to populations of different ethnic origin, given that ‘true’ causal variants may be located elsewhere.^8^ Difficulties in replicating signals can be due to the ‘lead’ polymorphism(s) identified in Europeans tagging smaller regions in individuals of African origin.^3–5^ ^9^ While transferability of ‘obesity-susceptibility’ loci to Asians is generally good, there is less consistency of findings with respect to populations of African ancestry.^26^ Interestingly, Domingue *et al*^22^ reported that alternate genetic risk scores derived from studies of populations of African ancestry performed similarly to ‘European risk scores’ in predicting BMI and obesity in African American young adults.

In summary, there appears to be evidence for shared genetic influences on weight status across diverse populations, including our sample of rural Gambians. Weaker effect sizes observed here, particularly in childhood, compared to those reported in populations of European origin could reflect differences in genetic architecture, age groups studied, as well as lower and more variable nutrition levels and variability. Our findings support a role for genetic obesity susceptibility on weight status across the whole spectrum of nutritional availability.^29^ The progressive increase in genetic effect on weight status in our population suggests that the underlying obesity-related mechanisms are not inherently limited by age or developmental stage. A possible explanation for the discordance between our findings and previous reports^15^ ^17^ ^24^ is that the apparent plateau in these genetic effects seen in Europeans at late adolescence/early adulthood might reflect reduced sensitivity to energy homeostatic mechanisms associated with attainment of adequate/high weight status.^5^ Larger multiethnic longitudinal studies will need to be conducted to evaluate in detail the genetic contribution especially to early growth faltering. This is particularly important in the context of the now well-accepted paradigm for the ‘Developmental Origins of Health and Disease’ (DOHaD), which states that optimising growth during early life is beneficial to life-long health.

## Supplementary Material

Web supplement
