# Supplementary material for: Progressive influence of body mass index-associated genetic markers in rural Gambians
Source: J Med Genet. 2015 Apr 28;52(6):375–80. doi: 10.1136/jmedgenet-2014-102784 (PMC4453496; doi:10.1136/jmedgenet-2014-102784)
Supplement: Web supplement [file jmedgenet-2014-102784-s1.pdf]

Supplementary File

Progressive influence of BMI-associated genetic markers in rural Gambians

Anthony J. Fulford[1], Ken K. Ong[2], Cathy E. Elks[2], Andrew M. Prentice[1], Branwen J. Hennig[1]

[1] MRC International Nutrition Group at LSHTM, UK & MRC Unit, The Gambia; Department of Epidemiology and Population Health, London School of Hygiene & Tropical Medicine, Keppel Street, London WC1E 7HT, UK.

[2] MRC Epidemiology Unit, University of Cambridge, School of Clinical Medicine, Box 285 Institute of Metabolic Science, Cambridge Biomedical Campus, Cambridge CB2 0QQ, UK.

Supplementary Table 1. Summary statistics of Gambian study population

A. Demographics	Females	Males	All
**Age [1], adults**			
N	930	496	1426
mean	26.3 y	28.9 y	
(range)	(20.0, 82.8 y)	(20.0, 82.6 y)	
**Village**			
Keneba	844	625	1469
Manduar	330	241	571
Kanton Kunda	294	201	495
**Ethnicity, all (N, %)**			
Mandinka	1418	1003	2421
Fula	36	44	80
Other	13	21	34
**B. Anthropometry at specific ages**	**Females**	**Males**	**All**
**Birth**			
N	730	673	1403
WT mean	2.87	3.03	
(range)	(1.40, 4.02)	(1.44, 4.41)	
zWT(UK)	-1.22	-1.12	
(range)	(-4.98, 1.31)	(-4.75, 1.71)	
**2 years [2]**			
N	1044	776	1820
WT mean	9.64	9.64	
(range)	(5.17, 14.64)	(5.71 , 13.89)	
HT mean	-2.14	-2.55	
(range)	(-8.33, 1.79)	(-7.55, 0.96)	
zWT(UK)	80.13	80.13	
(range)	(60.82, 99.09)	(60.34, 97.12)	
zHT(UK)	-1.83	-2.16	
(range)	(-8.05, 4.27)	(-8.57, 3.34)	
**Adult [3]**			
N	929	497	1426
WT mean	54.65	59.68	
(range)	(33.74, 100.02)	(35.92, 115.42)	
HT mean	-0.59	-1.39	
(range)	(-4.55, 3.28)	(-6.09, 3.25)	
zWT(UK)	159.10	170.57	
(range)	(137.40 179.40)	(148.60, 197.10)	
zHT(UK)	-0.75	-0.97	
(range)	(-4.35, 2.61)	(-4.12, 2.84)	
BMI mean	21.56	20.47	
(range)	(12.64, 37.16)	(11.67, 36.84)	

BW, birthweight; LG, length; HT, height; zHT(UK), height-for-age z-score; WT, weight; zWT(UK), weight-for-age z-score. **A.** [1] Analyses other than those of adults involved at least two age points from each individual, age across the whole study population (N=2535) is thus not shown. Further details on numbers per age group are shown in Table 1. **B.** zHT(UK) and zWT(UK) refer to z-scores derived from UK standards for comparative purposes (Freeman JV *et al.* Cross sectional stature and weight reference curves for the UK, 1990. Arch Dis Child 1995;73:17– 24). [2] nearest observation to 2 years of age (>1.5 and <2.5 y); [3] first measurement at adult age (>20 y).

Supplementary Table 2. Details of polymorphisms genotyped in Gambian study population

SNP ID	Gene	Effect allele	Other allele	Homo-zygotes for effect allele [N]	Hetero-zygotes [N]	Homo-zygotes for other allele [N]	Imputed genotypes [N]
rs10146997	*NRXN3*	G	A	610	1302	640	3
rs10838738	*MTCH2*	G	A	2467	86	2	0
rs10913469	*SEC16B*	C	T	1309	1042	197	7
rs11847697	*PRKD1*	T	C	914	1228	412	1
rs12016871	*MTIF3*	T	C	2373	172	6	4
rs12488483	*CADM2*	G	A	200	1071	1275	9
rs1514175	*TNNI3K*	A	G	169	1005	1364	17
rs1555543	*PTBP2*	C	A	737	1337	423	58
rs17782313	*MC4R*	C	T	1502	815	204	34
rs206936	*NUDT3*	G	A	389	1255	900	11
rs2112347	*FLJ35779*	T	G	587	1251	688	29
rs2241423	*MAP2K5*	G	A	470	1306	774	5
rs2287019	*QPCTL*	C	T	42	493	1946	74
rs2568958	*NEGR1*	A	G	427	1253	867	8
rs2890652	*LRP1B*	C	T	931	1230	373	21
rs3810291	*TMEM160*	A	G	2237	296	11	11
rs4929949	*RPL27A*	C	T	877	1224	451	3
rs6548238	*TMEM18*	C	T	50	532	1972	1
rs713586	*RBJ_POMC*	C	T	14	328	2181	32
rs7138803	*BCDIN3D_FAIM2*	A	G	1976	524	52	3
rs7640855	*CADM2*	A	G	2441	106	4	4
rs7647305	*TRA2B_ETV5*	C	T	459	1250	835	11
rs887912	*FANCL*	T	C	2287	257	9	2
rs925946	*BDNF*	T	G	1578	835	140	2
rs987237	*TFAP2B*	G	A	2010	509	29	7
rs989139	*GPRC5B*	C	T	503	1213	832	7
rs9941349	*FTO*	T	C	1920	578	52	5
rs4836133	*ZNF608*	A	C/G	31	516	2007	1
rs13107325	*SLC39A8*	T	C	2494	0	0	NA
rs10938397	*GNPDA2*	G	A	2538	0	0	NA

SNPs associated with BMI by Speliotes and colleagues (2010) or surrogate markers to the original SNPs, locating within the same haplotype block in HapMap Yoruba and Caucasians (r^2>0.8), were screened in our Gambian population (N=). Note: No suitable surrogate SNP was identified for KCTD15 rs11084753, LRRN6C rs2183825 and SH2B1 rs7498665.
